# Chemerin and Cancer

**DOI:** 10.3390/ijms20153750

**Published:** 2019-07-31

**Authors:** Oliver Treeck, Christa Buechler, Olaf Ortmann

**Affiliations:** 1 Department of Obstetrics and Gynecology, University Medical Center Regensburg, 93053 Regensburg, Germany; 2 Department of Internal Medicine I, University Medical Center Regensburg, 93053 Regensburg, Germany

**Keywords:** adipokine, chemerin, leukocyte, cancer

## Abstract

Chemerin is a multifunctional adipokine with established roles in inflammation, adipogenesis and glucose homeostasis. Increasing evidence suggest an important function of chemerin in cancer. Chemerin’s main cellular receptors, chemokine-like receptor 1 (CMKLR1), G-protein coupled receptor 1 (GPR1) and C-C chemokine receptor-like 2 (CCRL2) are expressed in most normal and tumor tissues. Chemerin’s role in cancer is considered controversial, since it is able to exert both anti-tumoral and tumor-promoting effects, which are mediated by different mechanisms like recruiting innate immune defenses or activation of endothelial angiogenesis. For this review article, original research articles on the role of chemerin and its receptors in cancer were considered, which are listed in the PubMed database. Additionally, we included meta-analyses of publicly accessible DNA microarray data to elucidate the association of expression of chemerin and its receptors in tumor tissues with patients’ survival.

## 1. Chemerin—A Multifunctional Cytokine and Adipokine

Chemerin, also known as retinoic acid receptor responder 2 (RARRES2), is expressed ubiquitously, but is most abundant in adipose tissue and the liver. Human chemerin is a 163 amino acid protein whose 20 amino acid N-terminal signal sequence is removed prior to secretion of the biologic inactive prochemerin into the bloodstream. Chemotactic active forms are produced by C-terminal processing. Extracellular serine and cysteine proteases generate different chemerin isoforms with chemerin 157 being the most active variant [1,2]. The biologic activities of chemerin isoforms are primarily mediated by two receptors, chemokine-like receptor 1 (CMKLR1) and G protein-coupled receptor 1 (GPR1) [3]. Chemerin similarly binds and activates both receptors, which triggers different cellular responses. Whereas activation of CMKLR1 leads to strong calcium mobilization and ERK1/2 phosphorylation, chemerin binding to GPR1 only leads to weak activation of both signaling mechanisms [4]. C-C motif chemokine receptor like 2 (CCRL2) is an additional chemerin receptor without known downstream signaling, which is thought to affect local activity of chemerin by presenting it to CMKLR1 and GPR1 [2].

Chemerin is a chemoattractant protein and its role in inflammation was studied in detail. In experimental pancreatitis chemerin infusion prior to disease induction reduces NF-κB signaling and thus exerts anti-inflammatory activities [5]. Chemerin activates NF-κB and up-regulates expression of adhesion molecules in endothelial cells thus enhancing monocyte adhesion and development of atherosclerosis [6]. In diabetic nephropathy chemerin activates the p38 MAPK pathway and thereby contributes to inflammation and renal injury [7]. In allergic asthma chemerin is shown to inhibit CCL2 secretion and subsequent recruitment of inflammatory dendritic cells [8]. Chemerin may be regarded as an immunoregulatory protein, and dependent on the context, acts as an anti-inflammatory or pro-inflammatory mediator.

Circulating chemerin is increased in obesity and may contribute to adiposity-related dyslipidemia, low-grade inflammation, hypertension and insulin resistance. Chemerin activation of CMKLR1 and GPR1 is not induced in parallel and short, inactive isoforms are elevated in the obese [9]. Detailed evaluation of chemerin processing and the activities of the different isoforms are essential to clarify the function of chemerin in obesity and its related comorbidities.

Chemerin protein levels are elevated in hypertensive patients, and experimental studies support a causative role of chemerin in the control of blood pressure [1]. Angiotensin-I-converting enzyme (ACE) cleaves angiotensin I to produce angiotensin II, a physiological regulator of blood pressure. Inhibition of ACE exerts antihypertensive effects and lowers serum and renal chemerin protein [10]. ACE removes two C-terminal amino acids from the decapeptide chemerin 145–154 indicating a function in chemerin processing [11]. Future work that evaluates the role of particularly the short isoforms of chemerin will help to establish the function of this chemokine in blood pressure control.

In the tightly controlled glucose metabolism, chemerin was shown to regulate glucose-induced insulin release and insulin-stimulated glucose uptake in skeletal muscle, but did not contribute to peripheral insulin resistance in general. Chemerin dose, duration of treatment, type of experimental model and the tissues/cell types analyzed seem to affect chemerin signaling [1,2]. Considering processing of chemerin to different isoforms, which vary in their biological effects and modulation of chemerin activity by the expression levels of its receptors, it is reasonable to postulate a complex association of chemerin with traits of the metabolic syndrome [1,2].

Adipose tissue growth in obesity includes adipocyte hyperplasia and hypertrophy [12]. Chemerin and CMKLR1 have been demonstrated to play a fundamental function in clonal expansion during early adipogenesis [13]. Knock-down of chemerin or CMKLR1 impairs 3T3-L1 cell adipogenesis [14]. Peroxisome proliferator-activated receptor γ (PPARγ) is a crucial regulator of adipogenesis and also elevates chemerin levels [13]. Chemerin release is strongly induced in hypertrophic adipocytes and this seems to contribute to higher systemic levels in the obese [15].

Recent studies suggested that chemerin plays an important role in cancer. Chemerin was found reduced or upregulated in cancer tissues and protective as well as promoting effects on carcinogenesis were identified. Thus similar to its role in inflammation, where chemerin acts as a pro- and anti-inflammatory factor, its effect in cancer depends on the disease context [16].

## 2. Chemerin and Cancer

### 2.1. Molecular Mechanisms Underlying the Role of Chemerin in Cancer

#### 2.1.1. Chemerin and Leukocyte Recruitment

Chemerin is an important chemoattractant inducing immunocyte recruitment by its receptors CMKLR1, GPR1 and CCRL2, leading to suppression of tumor growth. Activation of chemoattractant receptors triggers arrest, diapedesis and infiltration of specialized immune cells into the tumor microenvironment, regulating the growth and survival of cancer. Anti-tumoral effector immune cells can slow down the growth of malignancies, like immunostimulatory DC, NK cells and cytotoxic T cells. On the other hand, cancer cells can escape anti-tumoral immune responses in order to survive by different mechanisms like the recruitment of immunosuppressive regulatory T cells or myeloid-derived suppressor cells (MDSC), which are able to inhibit cytotoxic anti-tumor responses. The balance between pro- and anti-tumoral leukocytes finally determines tumor progression [17].

Chemerin receptors are expressed in normal and cancer tissues and on various immune cells. In an early study, CMKLR1 was found in human blood to be expressed by plasmacytoid dendritic cells (pDCs) only, but not in monocytes, neutrophils, eosinophils, lymphocytes or myeloid DCs [18]. A conflicting study identified CMKLR1 protein not only in all human pDCs, but also in about 40% of myeloid DCs, and reported expression of this receptor in mature DCs [19]. LPS and interferon (IFN)-γ were reported to enhance CMKLR1 transcription in macrophages. These inflammatory stimuli induced monocytes to differentiate to the so-called M1 macrophages. M1 but not M2 macrophages were chemotactic to chemerin [20]. Chemerin affects local inflammatory processes, which are characterized by the induction of pro-inflammatory as well as anti-inflammatory and pro-resolving factors, and thereby can accelerate its resolution [21].

Chemerin has been shown to promote pDC migration [18]. This subset of dendritic cells produces type I interferons and has a function in innate and adaptive immune response and cancer. In cancer, pDCs were reported to exert a decreased or absent IFN-α production and contribute to the establishment of an immunosuppressive tumor microenvironment [22]. Chemerin further was shown to promote chemotaxis of macrophages and monocyte derived immature dendritic cells generated by granulocyte macrophage colony stimulating factor (GM–CSF) and IL-13 [23]. A separate study found that chemerin attracted pDC but not mDC (derived from monocytes stimulated with GM–CSF and IL-4) [18]. Mature DCs can activate resting NK cells, which in turn either kill or enable maturation of immature DCs. Chemerin attracts NK cells and mediates co-localization of NK cells with pDC and mDC [24].

In a melanoma model, chemerin transfected cancer cells exhibited a growth inhibitory immune cell distribution in the tumor microenvironment, which was characterized by a higher number of NK and T cells and a relative decline of MDSC and pDCs. In vivo experiments showed that chemerin-expressing mouse B16F0 melanoma grew significantly more slowly than control tumors. Importantly, these tumor suppressive effects were specifically mediated by NK cells, as only NK cell depletion abrogated the effect, and required host expression of CMKLR1, as chemerin-expressing tumors showed accelerated growth in CMKLR1-negative mice [25]. With regard to adrenocortical carcinoma (ACC), studies report that chemerin is down-regulated in malignant tumors compared to benign or normal tissue [26,27]. These studies also report that increased chemerin serum levels were associated with better overall survival, and proposed that tumors may downregulate chemerin to escape immune defenses while host systems may up-regulate chemerin to activate immune responses. Supporting this hypothesis, mouse xenograft models confirmed that the increased serum chemerin levels do not result from expression in tumor tissue, but result of host secretion suggesting an important role of chemerin in host-mediated leukocyte recruitment [27]. Other studies showed that chemerin is expressed in endothelial cells being triggered by inflammatory cytokines, which resulted in increased dendritic cell transmigration mediated by CMKLR1 [28]. In mouse models chemerin has also been observed to suppress M2 macrophage polarization, but to increase the production of pro-inflammatory cytokines such as IFN-γ [29].

In conclusion, an important mechanism by which chemerin exerts anti-tumoral effects is recruiting growth inhibitory immune cells like NK cells to the tumor microenvironment. Tumor cells in turn are reported to reduce chemerin expression to escape immune defense [30].

#### 2.1.2. Intracellular Signaling of Chemerin Receptors

Expression of chemerin and its receptors has been detected in all tumor types tested, but their expression levels vary between different cancer entities and individual patients (Supplemental Figure S1) [31]. Using the GEPIA server analyzing RNAseq data of 9736 tumors and 8587 normal samples of the TCGA and GTEx projects revealed chemerin mRNA expression to be down-regulated in 23 of 31 cancer entities (74.2%), but to be up-regulated in eight of 31 cancer types tested (25.8%; Supplemental Figure S2) [32]. Chemerin has been reported to activate both tumor-promoting and -suppressing intracellular pathways in a receptor- and context-specific manner. Chemerin binding to CMKLR1, but not to GPR1 or CCRL2, strongly increases intracellular calcium concentration, decreases cyclic AMP levels and induces the phosphorylation of p42-p44 MAP kinases, through the Gi class of G proteins [4,23]. Binding of chemerin to CMKLR1 or GPR1 led to recruitment of β-arrestin 1 and 2 [4]. Arrestin binding to the receptors blocks further G protein-mediated signaling, targets receptors for internalization and redirects signaling to alternative G protein-independent pathways, such as β-arrestin signaling [33]. Binding of β-arrestin 1 or β-arrestin 2 exerts opposite effects in cancer progression by interacting with different signaling pathways, which may depend on the tumor microenvironment [34]. While β-arrestin 1 is reported to act tumor-promoting via interaction with c-Src [35], β-arrestin 2 has been shown to inhibit cancer growth and angiogenesis [36]. Chemerin receptor CCRL2 is considered to exert no specific downstream signaling activities, but to present chemerin to CMKLR1 and possibly to GPR1 [23,37,38]. A recent study reported that chemerin activates the transcriptional regulator serum-response factor (SRF) by binding to CMKLR1 and GPR1 through a RhoA and rho-associated protein kinase (ROCK)-dependent pathway [3]. SRF is a transcription factor, which has important roles in tumor progression [39,40]. Chemerin-triggered activation of SRF might be an important molecular mechanism underlying the role of chemerin in cancer. Induction of SRF by chemerin activated its target gene early growth response-1 (EGR1), a transcription factor, which has been suggested to be a tumor suppressor due to its growth inhibitory and pro-apoptotic effects [3,41,42]. Various studies report chemerin-triggered activation of c-FOS via CMKLR1 and SRF [3,43,44]. While c-FOS has been initially demonstrated to act as an oncogene, which is associated with tumor progression and decreased survival of cancer patients [45], recent studies also discovered a tumor-suppressing and pro-apoptotic function of c-FOS in various cancer types including ovarian cancer, hepatocellular carcinoma or prostate cancer and has been shown to be associated with increased survival in ovarian cancer [46,47,48,49,50]. Furthermore, a recent study demonstrated that chemerin suppresses hepatocellular carcinoma (HCC) metastasis through upregulation of expression and phosphatase activity of tumor suppressor PTEN by interfering with PTEN-CMKLR1 interaction, resulting in decreased p-Akt levels, also leading to suppressed migration, invasion and metastasis of HCC cells in vitro. Positive correlation between chemerin and PTEN were also observed in HCC clinical samples [51]. In vivo xenograft mouse models on adrenocortical cancer cells demonstrated that chemerin can decrease the levels of phosphorylated p38 MAPK and β-catenin and was suggested to act as a tumor suppressor [26]. Since the tumor-promoting role of Wnt/β-catenin and MAPK pathway activities is well established [52,53], chemerin might mediate tumor inhibition by reducing Wnt/β-catenin and MAPK pathway activity in adrenocortical carcinoma and, potentially, other cancer types.

In contrast, phosphorylation of p38 and ERK1/2 MAP kinases was reported to be elevated in gastric cancer cells after treatment with chemerin in vitro, leading to increased invasiveness of these cells and up-regulation of vascular endothelial growth factor (VEGF) and matrix metalloproteinase 7 (MMP-7) [54]. Given that MAPK pathways are known to enhance tumor progression and invasion in various cancer types, several other studies support the finding that chemerin may stimulate tumor growth via these mechanisms in gastric cancers [55,56]. Chemerin-triggered activation of MMP-7 expression is considered to be an important molecular mechanism underlying the increased invasiveness of gastric cancer cells, as this protease facilitates tumor cell invasion by degradation of extracellular matrix components, E-cadherin and integrins [57]. Chemerin-triggered activation of MMP expression and invasion of cancer cells has been reported in a variety of studies, including ones on squamous esophageal cancer and neuroblastoma [58,59], supporting the hypothesis that MMP activation is a mechanism underlying a tumor-promoting effect of chemerin in some tumor types.

**Figure 1:**
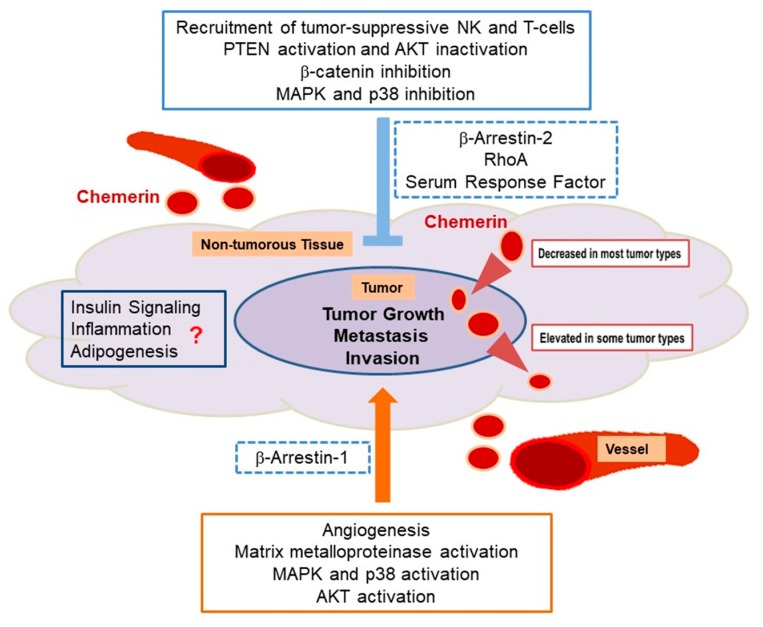
Anti-tumoral and cancer-promoting effects of chemerin. Anti-tumoral activities of chemerin, which are dominant in most cancer entities, include inhibition of MAPK, β-catenin and AKT where the latter is achieved by PTEN activation. These actions partially are mediated via the β2-arrestin and RhoA/serum response factor induced signaling. Both pathways are activated by chemerin binding to GPR1 and CMKLR1. In contrast, tumor-promoting effects are mediated by activation of matrix metalloproteinases, MAPK, p38 and AKT with involvement of β-arrestin-1. Angiogenesis is partly enhanced via these pathways. In about ¾ of the tumor types tested, chemerin expression is reduced in cancer tissue when compared to non-tumorous tissues, whereas in about ¼ of cancer entities chemerin expression is elevated. Insulin resistance, inflammation and adipogenesis are influenced by chemerin and may also contribute to tumor growth. Impact of these factors was not studied in detail so far.

In conclusion, with regard to intracellular chemerin receptor signaling, convincing evidence both for anti-tumoral actions (activation of PTEN, EGR1 and β-arrestin 2 and inhibition of β-catenin and MAPK activity) and tumor-promoting effects of chemerin (activation of MMP expression, p38 and ERK1/2 MAPK activity and of β-arrestin 1), were reported in different tumor types. Chemerin receptors are able to activate distinct signaling pathways, and chemerin seems to differentially regulate intracellular pathways depending on the tumor type. Thus, more effort is necessary to elucidate the role of chemerin receptor signaling in different cancer entities (Figure 1).

#### 2.1.3. Chemerin and Angiogenesis

The observed up-regulation of VEGF by chemerin leads to another important mechanism, which might cause a tumor-promoting effect of this adipokine, the activation of angiogenesis. Angiogenesis plays a central role in tumor growth, since solid cancers cannot grow beyond a limited size without an adequate blood supply. VEGF is considered as a key factor of angiogenesis, activating both migration and mitosis of endothelial cells and MMP expression. In a recent study, chemerin was demonstrated to be potently angiogenic via binding to CMKLR1 present on endothelial cells, and dose-dependently induced MMP-2 and MMP-9 activity in these cells [60]. These results were supported by a study observing the same effect of chemerin on angiogenesis and even more demonstrated that chemerin mediated the formation of blood vessels to a similar extent as VEGF [61]. Another recent study reported chemerin-triggered angiogenesis in mice. Chemerin also stimulated the differentiation of human umbilical vein endothelium cells (HUVECs) into capillary-like structures, promoted their proliferation, and functioned as a chemoattractant in migration assays. Chemerin promoted angiogenesis by phosphorylation of Akt and p42/44 extracellular signal-regulated kinase (ERK). Knockdown of chemerin receptor CMKLR1, but not of CCRL2, completely inhibited the chemerin-induced migration and angiogenesis of HUVECs, which indicated that chemerin promoted the migration and angiogenic activities mainly through CMKLR1 [62]. Taken together, convincing data clearly suggest that chemerin induces endothelial angiogenesis, and thereby is able to promote tumor growth.

### 2.2. Expression of Chemerin and its Receptors and Cancer Survival

#### 2.2.1. Breast Cancer

**Figure 2:**
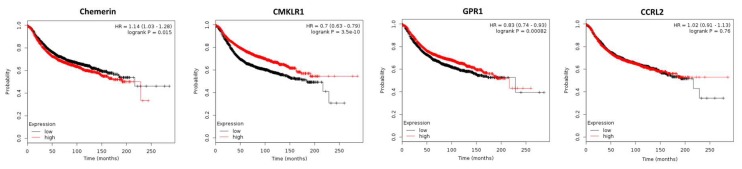
Kaplan–Meier diagrams showing the relapse-free survival (RFS) of 3951 breast cancer patients depending on expression of chemerin and its receptors CMKLR, GPR1 and CCRL2 in tumor tissue, based on a metaanalysis of DNA microarray data (http://kmplot.com/analysis/) [64].

The role of chemerin in breast cancer has been only addressed by a very limited number of studies. A recent study including 117 breast cancer patients reported that serum chemerin levels did not significantly differ between early or advanced-stage breast cancer [63]. Chemerin and its receptors have been shown to be expressed in breast cancer tissue by means of immunohistochemistry (IHC) or DNA microarray analysis [64]. In a recent study demonstrating CCRL2 expression in breast cancer by means of IHC, increased amounts of CCRL2 were found in breast tumor tissues with high immune cell infiltration. Its expression was upregulated in the presence of pro-inflammatory cytokines, IL-1β, TNF-α, IL-6 and especially IFN-γ [65]. With regard to chemerin expression in breast cancer tissue, a small study including 53 patients [66] detected a significantly higher expression of chemerin in malignant tissue than in the corresponding normal breast tissue (*p* = 0.001). Moreover, its expression was significantly higher in metastatic lymph nodes than in the primary tumor (*p* = 0.01). Chemerin expression was found to be weakly associated with tumor size (*r* = 0.235, *p* = 0.03), lymph node metastasis (*r* = 0.265, *p* = 0.045), distant metastasis (*r* = 0.267, *p* = 0.02) and showed good association with tumor grading, (*r* = 0.421, *p* = 0.004). Kaplan–Meier survival analysis revealed that patients with higher chemerin expression had worse overall survival in comparison to those with a lower chemerin expression, (*p* = 0.001). In contrast to this study on a small patients´ collective, in a metaanalysis of publicly available DNA microarray data of 3951 breast cancer patients, chemerin expression was observed to be significantly lower in breast cancer tissue than in a normal breast (*p* = 2.17 × 10^−7^) and use of Kaplan–Meier plotter software [64] revealed that high chemerin expression in breast cancer tissue did not affect overall survival (OS), but turned out to negatively affect relapse-free survival (RFS; *p* = 0.015; Figure 2). In contrast, high CMKLR1 expression, which did not significantly differ between normal and tumorigenic breast, had robust beneficial effects on RFS of breast cancer patients, but did not affect OS. Chemerin receptor GPR1 was found to be up-regulated in cancer tissue (*p* = 0.002), and high expression of GPR1 also led to prolonged RFS (*p* = 0.00082), but did not affect OS of breast cancer patients. Tissue expression of CCRL2 chemerin receptor, which was similar in normal and malignant breast tissue, neither affected RFS nor OS of women with breast cancer.

In conclusion, with regard to tumor expression of chemerin receptors, this adipokine is suggested to exert a tumor-suppressive role in breast cancer via binding to CMKLR1 and GPR1. DNA microarray data from 3951 breast cancer patients demonstrated that high mRNA expression of chemerin receptors CMKLR1 and GPR1 in tumor tissue was strongly associated with a longer RFS of breast cancer patients, whereas tissue expression of chemerin slightly decreased RFS and CCRL2 did not significantly affect patients’ survival. Given that tumor expression of chemerin is not the major source of this adipokine, the effects of CMKLR1 and GPR1 on survival in this context must be considered to be most relevant, as they bind chemerin from all sources and have been shown to exert intracellular downstream signaling, in contrast to CCRL2. The small IHC-based study including 53 patients mentioned above, which came to conflicting results, was judged to be less significant. The fact that the OS was not significantly affected in this 3951 patients collective might be due to the reported tumor-promoting effects of chemerin like activation of endothelial angiogenesis. However, further studies correlating chemerin serum levels and activity with survival are needed as well as further attempts to integrate the anti-tumoral and proposed tumor-promoting effects of this adipokine on survival of breast cancer patients.

#### 2.2.2. Ovarian Cancer

**Figure 3:**
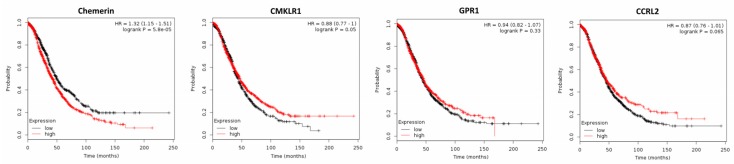
Kaplan–Meier diagrams showing the overall survival (OS) of 1656 ovarian cancer patients depending on expression of chemerin and its receptors CMKLR1, GPR1 and CCRL2 in tumor tissue, based on a metaanalysis of DNA microarray data (http://kmplot.com/analysis/) [71].

High levels of active chemerin have been found in a large proportion of ascitic fluids of ovarian carcinomas [67]. Bioactive chemerin and its receptor CMKLR1 have also been detected in human granulosa cells [68]. A recent study demonstrated expression of CMKLR1 in a granulosa cell tumor cell line to be higher than in epithelial cancer cells, whereas chemerin expression and secretion were lower. Treatment with chemerin in vitro was reported not to affect growth of ovarian non-cancer and cancer cell lines [69]. Using an obesity mouse model, the chemerin/CMKLR1 system was observed to be upregulated in the serum, ovaries and granulosa cells and was associated with apoptotic ovarian follicles, oxidative stress and apoptosis biomarkers. Further in vitro experiments confirmed the apoptotic effect of chemerin on granulosa cells [70]. No publications are available examining the effect of serum chemerin or its tumor expression on ovarian cancer growth. However, analyzing publicly available DNA microarray data of 1656 ovarian cancer patients revealed a lower chemerin expression in ovarian cancer tissue than in normal ovary (*p* = 0.018), and using the Kaplan–Meier plotter software revealed that high chemerin expression negatively affected both OS (*p* = 5.8 × 10^−5^) and progression-free survival (PFS; *p* = 0.00024) of ovarian cancer patients [64,71]. In contrast, higher expression of chemerin receptor CMKLR1 had a beneficial effect both on OS (*p* = 0.05; Figure 3) and on PFS (*p* = 0.0009). GPR1, which was down-regulated in ovarian cancer tissue (*p* = 0.006), had no significant effect on OS or on PFS of ovarian cancer patients. Expression of CCRL2, which was similar in normal and cancer tissue, also did not affect OS or PFS of ovarian cancer patients (Figure 3 and data not shown).

In conclusion, with regard to tumor expression of chemerin receptors, this adipokine is suggested to exert a tumor-suppressive role in ovarian cancer via binding to CMKLR1 and CCRL2. The observation that chemerin was able to induce apoptosis in ovarian follicles via CMKLR1 raises the question, whether the same effect could be present in ovarian cancer. The results of DNA microarray analyses of 1656 ovarian cancer patients, demonstrating a significantly decreased survival of patients with high chemerin tumor expression, seemed not to support this hypothesis, but it has to be considered that tumor tissue is only a minor source of chemerin. The prolonged OS of patients with high expression of CMKLR1 and CCRL2 clearly suggested an anti-tumoral effect of serum chemerin being activated in ovarian cancer tissue. However, further studies, particularly on serum chemerin levels, on protein expression of its receptors in ovarian cancer tissue and considering the reported tumor-promoting angiogenic effects of this adipokine, are required to further elucidate the role of chemerin in ovarian cancer.

#### 2.2.3. Non-Small-Cell Lung Cancer (NSCLC)

Circulating chemerin has been reported to be elevated and to exert adverse effects in non-small-cell lung cancer (NSCLC) patients. In a recent case-control study including 220 patients and controls, NSCLC cases exhibited significantly elevated serum chemerin levels compared to controls. In NSCLC cases, chemerin was positively associated with tumor and inflammatory biomarkers, number of infiltrated lymph nodes and NSCLC stage. Serum chemerin was found to be independently associated with NSCLC [72]. These results were supported by another large study reporting elevated levels of circulating chemerin in NSCLC patients, and higher levels of chemerin being associated with advanced TNM stage, lymph node metastasis and distant metastasis. Further analyses revealed that the higher serum chemerin patients had a shorter OS and PFS compared with lower chemerin patients and identified serum chemerin to be an independent risk factor for the prognosis of NSCLC patients [73]. Increased chemerin serum levels in NSCLC patients were also reported in a smaller study, which could not find any association with clinicopathological parameters [74]. A recent study analyzing chemerin expression in NSCLC tissue by IHC in 108 patients observed a decreased expression of this adipokine in about half of the tested patients. Chemerin expression was significantly correlated with the histological grade and the infiltration of NK cells. NSCLC patients with a lower chemerin expression had poorer survival rates than those with a higher expression. Multi-variable Cox regression analysis suggested expression of chemerin to be an independent predictor of a better prognosis for patients with NSCLC [75].

**Figure 4:**
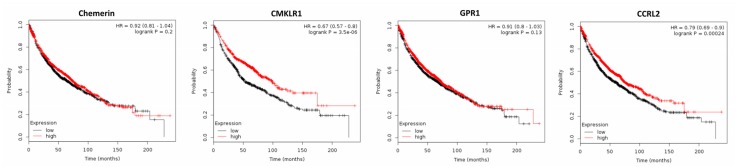
Kaplan–Meier diagrams showing the overall survival (OS) of 1926 patients with non-small-cell lung cancer (NSCLC) depending on expression of chemerin and its receptors CMKLR1, GPR1 and CCRL2 in NSCLC tissue, based on a metaanalysis of DNA microarray data (http://kmplot.com/analysis/) [76].

Analyzing publicly available DNA microarray data of 1926 NSCLC patients revealed that chemerin expression was significantly down-regulated in lung cancer tissue when compared to normal lung (*p* = 0.0009), supporting the results of the study mentioned above [76]. However, in contrast to this study, the use of Kaplan–Meier plotter software on these data revealed that high chemerin expression did not affect OS nor time to first progression (FP; Figure 4 and data not shown). CMKLR1, which was found to be up-regulated in cancer tissue (*p* = 0.005), positively affected OS of NSCLC patients (*p* = 3.5 × 10^−6^), but not FP. Expression of GPR1 was down-regulated in NSCLC tissue (*p* = 7.25 × 10^−9^) and did not affect patients´ OS or FP. Elevated expression of CCRL2 was found to have a positive effect on OS and FP of NSCLC patients (*p* = 0.0002 or *p* = 0.016, respectively; Figure 4 and data not shown).

Importantly, a study performing a genome-wide scan of 307,260 single-nucleotide polymorphisms (SNPs) in 327 advanced-stage NSCLC patients revealed that only a CMKLR1 SNP was significantly associated with overall survival [77].

In conclusion, chemerin serum levels have been reported to be elevated in NSCLC patients, to exert adverse effects on survival and to be an independent risk factor of prognosis of NSCLC patients. In contrast, gene expression analysis of 1926 NSCLC patients revealed that tumor expression of this adipokine did not affect survival. In conflict with the above mentioned studies reporting adverse effects of serum chemerin, the metaanalysis of DNA microarray data demonstrated tumor expression of CMKLR1 and CCRL2 to exert significant beneficial effects on the OS of NSCLC patients, a fact that was supported for CMKLR1 by SNP analyses. To address this controversy, further studies are needed examining chemerin receptor activation in tumor tissue and their expression on the protein level to validate the role of chemerin receptors in NSCLC. It also has to be examined to what extent chemerin-triggered endothelial angiogenesis might contribute to the reported adverse effects of serum chemerin in this cancer entity.

#### 2.2.4. Gastric Cancer

**Figure 5:**
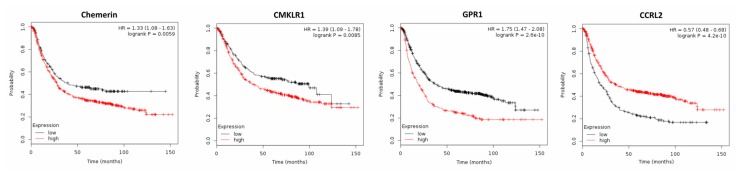
Kaplan–Meier diagrams showing the overall survival (OS) of 876 patients with gastric cancer depending on expression of chemerin and its receptors CMKLR1, GPR1 and CCRL2 in gastric cancer tissue, based on a metaanalysis of DNA microarray data (http://kmplot.com/analysis/) [80].

Two recent studies demonstrated elevated chemerin serum levels in patients with gastric cancer, which were associated with tumor progression. In the first study, the increase of serum chemerin levels was shown to be associated with elevated cellular invasiveneness, advanced clinical stages and non-intestinal type of gastric cancer [54]. These observations were supported by a second study reporting increased levels of circulating chemerin in gastric cancer patients, which also identified this adipokine as an independent predictor for five-year mortality (odds ratio (OR), 2.718; *p* = 0.005) and adverse event (OR, 2.982; *p* = 0.003) of gastric cancer. High plasma chemerin levels also were observed as an independent predictor for shorter OS and RFS and were suggested to be a potential prognostic biomarker in gastric cancer survival [78]. Chemerin receptors CMKLR1 and GPR1 have been recently reported to be expressed in gastric cancer tissue, as assessed by IHC. In a gastric cancer cell line, chemerin stimulated both, cellular migration and invasion, in a CMKLR1- and GPR1-dependent manner [79]. In line with these studies, the analysis of publicly available DNA microarray data of 876 gastric cancer patients by means of the Kaplan–Meier plotter software [80] demonstrated that high chemerin tumor expression reduced OS of gastric cancer patients (*p* = 0.0059; Figure 5) and also higher tissue expression of CMKLR1 and GPR1 significantly decreased OS of gastric cancer patients (*p* = 0.0085 or *p* = 1.7 × 10^−7^, respectively). In contrast, elevated expression of CCRL2 increased OS of these patients (*p* = 4.2 × 10^−10^).

In conclusion, all present publications and gene expression data clearly suggest a tumor-promoting effect of chemerin in gastric cancer. Both high chemerin serum levels and tumor expression were associated with shorter OS of gastric cancer patients. Chemerin receptors CMKLR1 and GPR1 induced migration and invasion of gastric cancer cells in vitro and were associated with a significantly decreased OS of gastric cancer patients. Thus, serum chemerin levels and tumor expression of CMKLR1 and GPR1 might have the potential to act as prognostic biomarkers in gastric cancer survival. However, further studies are needed to examine chemerin receptor expression on the protein level, their activation and to elucidate the mechanisms underlying the divergent effect of CCRL2.

#### 2.2.5. Hepatocellular Carcinoma

Hepatocellular carcinoma (HCC) is linked to inflammation and immunosuppression. Chemerin is highly expressed in the liver and implicated in the regulation of inflammation. However, the role of chemerin in HCC remains unclear. A recent study reported that chemerin was significantly decreased in blood and tumor tissues of HCC patients, and low tumor chemerin expression was associated with a bad prognosis [81]. Accordingly, two further studies suggested an anti-tumoral effect of chemerin in HCC. The first one also reported chemerin to be decreased in HCC tissue, and lower chemerin expression positively correlated with tumor size and the infiltration of DC and NK cells. Survival analysis indicated that HCC patients with lower chemerin expression had poorer survival than those with higher expression (*p* < 0.001). Multivariable Cox regression analysis revealed that the chemerin expression level was an independent factor for prognosis (HR 3.034, *p* = 0.047) [82]. In line with this study, decreased tumor expression of chemerin was also found to be associated with a poor prognosis of HCC patients in a second study. Additionally, administration of chemerin effectively suppressed extrahepatic and intrahepatic metastases of HCC cells, resulting in prolonged survival of tumor-bearing nude mice [51].

**Figure 6:**
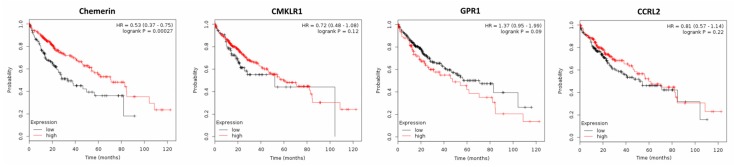
Kaplan–Meier diagrams showing the overall survival (OS) of 364 patients with hepatocellular carcinoma (HCC) depending on expression of chemerin and its receptors CMKLR1, GPR1 and CCRL2 in tumor tissue, based on a metaanalysis of DNA microarray data (http://kmplot.com/analysis/) [83].

Supporting the mentioned studies suggesting a tumor-suppressive role of chemerin, the metaanalysis of publicly available gene expression data of 364 patients with HCC by means of the Kaplan–Meier plotter software [83] revealed that high chemerin expression in cancer tissue significantly increased patients’ OS (*p* = 0.00027) and progression-free survival (PFS; *p* = 0.012). Higher expression of CMKLR1 did not significantly affect OS of HCC patients, but increased their PFS (*p* = 0.0017). Tumor expression of GPR1 or CCRL2 did not significantly alter OS or PFS of patients with HCC (Figure 6 and data not shown).

In conclusion, the majority of the present data clearly suggest an anti-tumoral role of chemerin in HCC. However, further studies are needed to elucidate the relationship of serum chemerin levels and cancer survival, to examine chemerin receptor activation and their tumor expression on the protein level.

#### 2.2.6. Other Cancer Types

In adrenocortical carcinoma (ACC), chemerin has been suggested to act as a tumor-suppressor. In a genome-wide gene expression study on 85 patients, chemerin expression was found to be strongly down-regulated in adrenocortical carcinoma when compared to benign tumors and was suggested to have an excellent diagnostic accuracy for distinguishing benign from malignant adrenocortical tumors [84]. Another study reported decreased tumor chemerin gene expression, but increased serum levels of this adipokine as compared with patients with benign adrenocortical tumors. Higher serum chemerin levels were associated with improved overall survival [27]. The decreased chemerin expression in ACC was demonstrated to be the result of chemerin gene CpG hypermethylation. In contrast, chemerin overexpression in ACC cell lines not only reduced cell proliferation, cell invasion and tumorigenicity in vitro, but also inhibited tumor growth in vivo in immunodeficient mouse xenograft models [26].

In acute myeloid leukemia (AML), a recent study also suggests a tumor-suppressing role of chemerin, demonstrating that chemerin was down-regulated in the bone marrow mononuclear cells of AML patients compared to that of healthy controls. In patients with AML, low chemerin expression correlated with poorer overall survival. It was shown that chemerin was independently able to prognosticate AML patients, and high chemerin expression was associated with positive prognosis [85]. Chemerin receptor CCRL2 was reported to be overexpressed in AML cells and was suggested to be a potential therapeutic target [86].

In melanoma, chemerin was shown to inhibit tumor growth by eliciting antitumor responses and altering the tumor microenvironment in favor of growth inhibition. Chemerin was found to be down-regulated in melanoma and high chemerin mRNA expression in tumors correlated with improved outcome in human melanoma. The anti-tumoral effect of chemerin was associated with increased recruitment of NK cells and was CMKLR1-dependent [25].

Further research is needed to elucidate the role of chemerin in cancer, particularly with regard to cancer entities not mentioned here.

## 3. Conclusions

Chemerin is a pleiotropic protein, which has been demonstrated to affect tumor growth, being able to exert both anti-tumoral and tumor-promoting effects. The majority of present data report down-regulation of chemerin in cancer tissue and suggest a tumor-suppressing role of chemerin in most cancer entities, being mediated by recruiting innate immune defenses and by growth-inhibitory downstream signaling by chemerin receptors CMKLR1 and GPR1. However, this anti-tumoral effect seems to be tissue-specific, since e.g., in gastric cancer, all available data suggest a tumor-promoting role of chemerin, which is mediated by different receptor signal transduction. Although the activating effect of this protein on endothelial angiogenesis is an important mechanism promoting tumor growth, it does not seem to significantly affect the beneficial role of chemerin. Association of tumor expression of chemerin receptors on patients’ survival was observed in most cancer entities. Further studies are needed to elucidate the role of this protein in different cancer types and to what extent therapeutic modulation of chemerin might be an option for cancer therapy.

## Supplementary Materials

Supplementary materials can be found at https://www.mdpi.com/1422-0067/20/15/3750/s1. Figure S1. Tumor tissue expression of chemerin and its receptors as assessed by means of RNA-seq shown as median FPKM (number Fragments Per Kilobase of exon per Million reads), generated by The Cancer Genome Atlas (TCGA). Data are from the open-access database (www.proteinatlas.org) [31]. The links to the single genes are: https://www.proteinatlas.org/ENSG00000106538-RARRES2/pathology, https://www.proteinatlas.org/ENSG00000174600-CMKLR1/pathology, https://www.proteinatlas.org/ENSG00000183671-GPR1/pathology and https://www.proteinatlas.org/ENSG00000121797-CCRL2/pathology. Figure S2. Comparison of chemerin mRNA expression in normal and tumor tissues using GEPIA, a newly developed interactive web server for analyzing the RNA sequencing expression data of 9736 tumors and 8587 normal samples from the TCGA and the GTEx projects [32]. (http://gepia.cancer-pku.cn/detail.php?gene=RARRES2). Values are expressed in transcripts per million (TPM). Abbreviations: ACC Adrenocortical carcinoma, BLCA Bladder Urothelial Carcinoma, BRCA Breast invasive carcinoma, CESC Cervical squamous cell carcinoma and endocervical adenocarcinoma, CHOL Cholangio carcinoma, COAD Colon adenocarcinoma, DLBC Lymphoid Neoplasm Diffuse Large B-cell Lymphoma, ESCA Esophageal carcinoma, GBM Glioblastoma multiforme, HNSC Head and Neck squamous cell carcinoma, KICH Kidney Chromophobe, KIRC Kidney renal clear cell carcinoma, KIRP Kidney renal papillary cell carcinoma, LAML Acute Myeloid Leukemia, LGG Brain Lower Grade Glioma LIHC Liver hepatocellular carcinoma, LUAD Lung adenocarcinoma, LUSC Lung squamous cell carcinoma, MESO Mesothelioma, OV Ovarian serous cystadenocarcinoma, PAAD Pancreatic adenocarcinoma, PCPG Pheochromocytoma and Paraganglioma, PRAD Prostate adenocarcinoma, READ Rectum adenocarcinoma, SARC Sarcoma, SKCM Skin Cutaneous Melanoma, STAD Stomach adenocarcinoma, TGCT Testicular Germ Cell Tumors, THCA Thyroid carcinoma, THYM Thymoma, UCEC Uterine Corpus Endometrial Carcinoma, UCS Uterine Carcinosarcoma. UVM Uveal Melanoma.
