# Supplementary material for: Chemerin and Cancer
Source: Int J Mol Sci. 2019 Jul 31;20(15):3750. doi: 10.3390/ijms20153750 (PMC6695761; doi:10.3390/ijms20153750)
Supplement: Supplementary file 1 [file ijms-20-03750-s001.pdf]

International Journal of
Molecular Sciences

MDPI

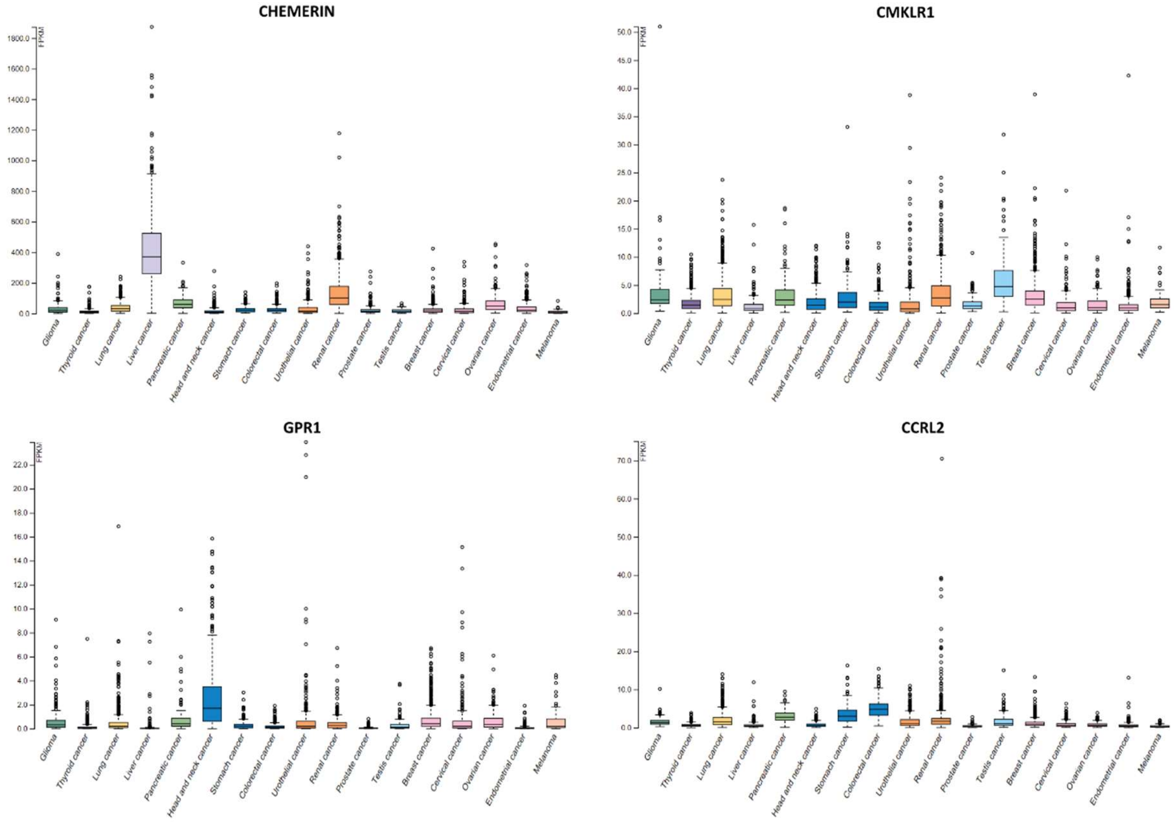

Figure S1. Tumor tissue expression of chemerin and its receptors as assessed by means of RNA-seq shown as median FPKM (number Fragments Per Kilobase of exon per Million reads), generated by The Cancer Genome Atlas (TCGA). Data are from the open-access database (www.proteinatlas.org) [31]. The links to the single genes are: https://www.proteinatlas.org/ENSG00000106538-RARRES2/pathology, https://www.proteinatlas.org/ENSG00000174600-CMKLR1/pathology, https://www.proteinatlas.org/ENSG00000183671-GPR1/pathology and https://www.proteinatlas.org/ENSG00000121797-CCRL2/pathology.

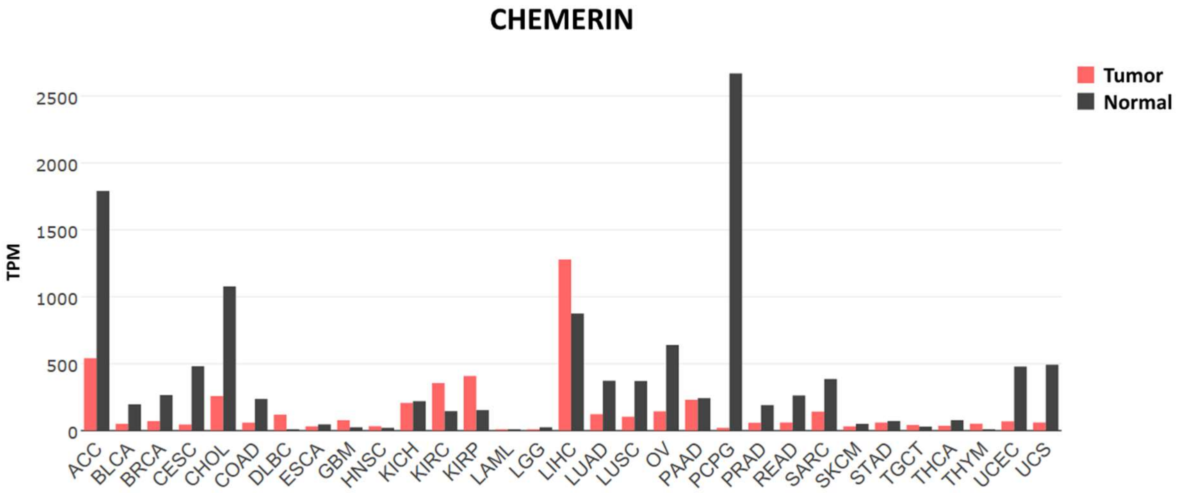

Figure S2. Comparison of chemerin mRNA expression in normal and tumor tissues using GEPIA, a newly developed interactive web server for analyzing the RNA sequencing expression data of 9736 tumors and 8587 normal samples from the TCGA and the GTEx projects [32]. (http://gepia.cancer-pku.cn/detail.php?gene=RARRES2). Values are expressed in transcripts per million (TPM).

Abbreviations: ACC Adrenocortical carcinoma, BLCA Bladder Urothelial Carcinoma, BRCA Breast invasive carcinoma, CESC Cervical squamous cell carcinoma and endocervical adenocarcinoma, CHOL Cholangio carcinoma, COAD Colon adenocarcinoma, DLBC Lymphoid Neoplasm Diffuse Large B-cell Lymphoma, ESCA Esophageal carcinoma, GBM Glioblastoma multiforme, HNSC Head and Neck squamous cell carcinoma, KICH Kidney Chromophobe, KIRC Kidney renal clear cell carcinoma, KIRP Kidney renal papillary cell carcinoma, LAML Acute Myeloid Leukemia, LGG Brain Lower Grade Glioma LIHC Liver hepatocellular carcinoma, LUAD Lung adenocarcinoma, LUSC Lung squamous cell carcinoma, MESO Mesothelioma, OV Ovarian serous cystadenocarcinoma, PAAD Pancreatic adenocarcinoma, PCPG Pheochromocytoma and Paraganglioma, PRAD Prostate adenocarcinoma, READ Rectum adenocarcinoma, SARC Sarcoma, SKCM Skin Cutaneous Melanoma, STAD Stomach adenocarcinoma, TGCT Testicular Germ Cell Tumors, THCA Thyroid carcinoma, THYM Thymoma, UCEC Uterine Corpus Endometrial Carcinoma, UCS Uterine Carcinosarcoma. UVM Uveal Melanoma.

© 2019 by the authors. Submitted for possible open access publication under the terms and conditions of the Creative Commons Attribution (CC BY) license (http://creativecommons.org/licenses/by/4.0/).
